# Impact of Thermal Fluctuations on Logarithmic Corrected Massive Gravity Charged Black Hole

**DOI:** 10.3390/e23101269

**Published:** 2021-09-28

**Authors:** Abdul Jawad, Shahid Chaudhary, Kazuharu Bamba

**Affiliations:** 1 Department of Mathematics, Lahore Campus, COMSATS University Islamabad, Lahore 54000, Pakistan; abduljawad@cuilahore.edu.pk (A.J.); shahidpeak00735@gmail.com (S.C.); 2 Division of Human Support System, Faculty of Symbiotic Systems Science, Fukushima University, Fukushima 960-1296, Japan

**Keywords:** massive gravity, helmholtz free energy, gibbs free energy, enthalpy, stability, hessian matrix

## Abstract

We investigate the influence of the first-order correction of entropy caused by thermal quantum fluctuations on the thermodynamics of a logarithmic corrected charged black hole in massive gravity. For this black hole, we explore the thermodynamic quantities, such as entropy, Helmholtz free energy, internal energy, enthalpy, Gibbs free energy and specific heat. We discuss the influence of the topology of the event horizon, dimensions and nonlinearity parameter on the local and global stability of the black hole. As a result, it is found that the holographic dual parameter vanishes. This means that the thermal corrections have no significant role to disturb the holographic duality of the logarithmic charged black hole in massive gravity, although the thermal corrections have a substantial impact on the thermodynamic quantities in the high-energy limit and the stability conditions of black holes.

## 1. Introduction

The thermodynamics of black holes (BHs) has a major role in BH physics and has gained much attention after the detection of Hawking radiations [1]. Bardeen [2] developed four laws of BH thermodynamics and explained the relationship between the thermodynamics of BH and gravity. This relationship was established by making proportionality between the temperature and surface gravity as well as entropy and surface area of the event horizon. In BH thermodynamics, Bekenstein’s area law yields entropy, while the first law of thermodynamics provides the temperature of BH [3,4]. After the discovery of these thermodynamical quantities of BHs, the thermodynamics of BH has become an important part for characterizing the physicality and viability of many BH solutions. The corrected thermodynamics of BH has important features, which bring local and global stability or instability, criticality, holographic duality and many other important aspects of BHs. The thermodynamical corrections due to fluctuations have gained a prominent place in BH physics. The corrected BH thermodynamics in the background of matter field were discussed in [5,6]. The influence of thermal fluctuations on charged anti-de Sitter (AdS) BH has shown excellent results of corrections in AdS BHs [7]. The deep thermodynamical analysis of BHs showed that the quantum approach at small scales to the thermodynamics of BH is inevitable, and it leads to corrections in various thermodynamical quantities. The GUP-corrected thermodynamics for all black objects is one of the important approaches [8]. The logarithmic corrections to Godel BH and quantum corrections to the thermodynamics of BH with the Cardy formula were also studied in [9,10], respectively. Nozari et al. [11] provided a detailed study of the influence of corrections on the BH thermodynamics. The quantum corrections to thermodynamics of quasi-topological BHs were studied in [12]. These thermal corrections for Schwarzschild–Beltrami–de Sitter BH [13] and the BH in MG were discussed in literature works [14]. The pioneering work of Frolov et al. [15] studied the quantum corrections in the BH thermodynamics. The correction terms alter the entropy area relationship, and it can be written as $S=S_{o}+\xi \log A+\eta_{1}A^{-1}+\eta_{2}A^{-2}\ldots$, where ξ, $\eta_{1}$, $\eta_{2}$, ..., are coefficients that depend upon the parameters of the BHs. [16]

Einstein’s theory of general relativity (GR) predicts the presence of the massless spin 2 particles known as gravitation [17]. Remarkable progress has been made in understanding the properties of massive gravity (MG) in four dimensions over the last few years [18]. Fierz and Pauli developed the first massive theory of gravity by connecting the terms of interaction to the linearized GR [19]. Dam et al. [20] found a singularity in the Newtonian potential in the massless limit of Fierz and Pauli massive theory of gravity. A well-known ghost-free nonlinear theory of MG was proposed by de Rham–Gabadadze–Tolley (dRGT) [21]. Due to the complexity of equations, it is not an easy task to find exact solutions of this model. However, it has been an attracting and motivated field of research in theoretical physics [22,23,24]. The significance of MG is not restricted to the non-perturbative analysis of gravity or BH solutions. It is also helpful for the study of cosmic acceleration of universe [25,26,27]. According to the Vainshtein mechanism, at small scales MG is the same as GR, while at large distances, it amends gravity [28]. Bamba et al. [29] studied the trace-anomaly driven inflation in f(T) gravity and in minimal massive bigravity. They also discovered the influence of the trace anomaly on inflation. Bamba et al. [30] presented non-minimal coupling in the (extended) nonlinear MG theories and showed that there is no viable thermal history of the universe in this case.

Recently, the issue of dark energy and modified gravity theories has attracted the intention of many researchers. Padmanabhan [31] discussed dark energy and gravity and provided useful results on dark energy by using the cosmological constant. Copeland et al. [32] studied the techniques that have been used to explain the observation of accelerating universe. In their work, they provided a number of dark energy models in addition to the conventional cosmological constant, K-essence and tachyon, etc. Durrer and Maartens [33] worked on dark energy and dark gravity. They reviewed the models that deals with dark energy problem within general relativity. Clifton et al. [34] presented a comprehensive study of recent work on modified theories of gravity and their cosmological consequences. K. Bamba et al. [35] explained the different dark energy cosmologies. They presented the ΛCDM cosmology, Little Rip and Pseudo-Rip universes, phantom and quintessence cosmologies with Types I, II, III and IV finite-time future singularities and non-singular dark energy universes. Capozziello and Laurentis [36] studied the basic principles for the gravitational theory and provided the geometrical interpretation to highlight the basic assumptions of general relativity and its possible extensions. Nojiri and Odintsov [37] provided the unified cosmic history in modified gravity from f(R) theory to Lorentz non-invariant models. Nojiri et al. [38] explained some problems of modified gravity in cosmology, emphasizing on inflation, bouncing cosmology and the late-time acceleration era. Sotiriou and Faraoni [39] reviewed f(R) theories in detail and presented all known formalisms. Felice and Tsujikawa [40] studied applications of f(R) theories to cosmology and gravity, such as inflation, dark energy, local gravity constraints, etc. Joyce et al. [41] identified the guiding principles for rigorous and consistent modifications of the standard model, and discussed the prospects for empirical tests. Bamba and Odintsov [42] studied the inflationary cosmology in modified gravity with its extensions in order to generalize the Starobinsky inflation model. Cai et al. [43] evaluated the cosmological solutions arising from f(T) gravity, both at the background and perturbation levels.

In 1930, Born and Infeld [44] constructed the first nonlinear electrodynamics (NED) theory, and it played the central role in D-brane physics. Different efforts have been made in the literature to develop the BI NED Lagrangian. The two well-renowned constructed NED are Logarithmic (LNE) and Exponential (ENE) [45,46]. LNE is used to remove divergence in the electric field, while the ENE makes it weaker than Maxwell theory. LNE has been discussed in various studies. For instance, BH solutions in Einstein–dilaton theory and their thermodynamics were discussed in [47]. AdS-dilaton BH in the presence of LNE and Lifshitz-dilaton BHs was discussed in [48,49].

The paper is structured as follows. In the next section, we present thermal corrections of logarithmic charged BH in MG. In Section 3, we discuss the holographic duality and thermal stability. In Section 4, we summarize our results in concluding remarks.

## 2. Thermal Corrections of Logarithmic Charged Black Hole in Massive Gravity

The (n+1) dimensional action of Einstein MG with negative cosmological constant in context of LNED is given by the following [50]:

(1) $$\begin{array}{c} S=\frac{1}{16\pi }\int D^{n+1}x\sqrt{-g}\left[R-2\Lambda -8\beta^{2}\ln\left(1+\frac{F}{8\beta^{2}}\right)+m^{2}\sum c_{i}U_{i}\left(g,\Gamma \right)\right], \end{array}$$

where R and $\Lambda =\frac{-n(n-1)}{2l^{2}}$ are the Ricci scalar and the cosmological constant, with *l* being the AdS space time radius, and $F=F_{\mu \nu }F^{\mu \nu }$ represents the Maxwell invariant. β is a nonlinear constant; for β→∞, the LNED changes to linear Maxwell. $m^{2}$ is a positive parameter of MG, and (m→0) represents the translation invariance. In Lagrangian (1), $c_{i}$, U and Γ are the constants, symmetric polynomials and reference metric, respectively. We consider the line element of (n+1) dimensional space time to obtain the static charged BH solution.

(2) $$\begin{array}{c} dS^{2}=-\psi \left(r\right)dt^{2}+\psi {\left(r\right)}^{-1}dr^{2}+r^{2}\left(d\theta^{2}+sin^{2}\theta d\phi^{2}\right). \end{array}$$

The metric of BH in context of LNED turns out to be as follows [47]:

(3) $$\begin{array}{ccc} \psi (r) & = & k-\frac{m_{0}}{r^{n-2}}-\frac{2r^{2}\Lambda }{n(n-1)}+\frac{8\beta^{2}r^{2}}{n(n-1)}\left(\ln\left(\frac{1+\Upsilon }{2}\right)+\frac{\left(2n-1)(1-\Upsilon \right)}{n}\right)\\ & + & \frac{8\left(n-1\right)q^{2}r^{4-2n}}{n^{2}\left(n-2\right)}F\left(\frac{1}{2},\frac{n-2}{2(n-1)},\frac{4-3n}{2-2n},\frac{-q^{2}r^{2-2n}}{\beta^{2}}\right)+\frac{c_{0}m^{2}r}{n-1}(c_{1}\\ & + & \frac{\left(n-1\right)c_{0}c_{2}}{r}+\frac{\left(n-1\right)\left(n-2\right)c_{0}^{2}c_{3}}{r^{2}}+\frac{\left(n-1\right)\left(n-2\right)\left(n-3\right)c_{0}^{3}c_{4}}{r^{2}}), \end{array}$$

where $\Upsilon =\sqrt{1+\frac{q^{2}}{\beta^{2}}r^{2-2n}}$, **F** is hypergeometric function and $m_{0}$ is related to the total mass of the BH. *q* is a constant, which represents the total charge of BH.

In order to find the mass of logarithmic charged BH, setting $\psi (r_{+})=0$ in Equation (2), one can obtain the following relation:

(4) $$\begin{array}{ccc} m_{0} & = & kr_{+}^{n-2}+\frac{8\beta^{2}r_{+}^{n}}{n(n-1)}(\ln\frac{\left(1+\sqrt{1+\frac{q^{2}}{\beta^{2}}r^{2-2n}}\right)}{2}+(\left(2n-1\right)(1-(1+\frac{q^{2}}{\beta^{2}}\\ & \times & r^{2-2n}{)}^{\frac{1}{2}}{))\left(n\right)}^{-1})-\frac{2r_{+}^{n}\Lambda }{n(n-1)}+\frac{8\left(n-1\right)q^{2}r_{+}^{-2n}}{n^{2}\left(n-2\right)}F(\frac{1}{2},\frac{n-2}{2(n-1)},(4\\ & - & {3n)\left(2-2n\right)}^{-1},\frac{-q^{2}r_{+}^{2-2n}}{\beta^{2}})+\frac{c_{0}m^{2}r_{+}^{n-1}}{n-1}(c_{1}+\frac{\left(n-1\right)c_{0}c_{2}}{r_{+}}+((n\\ & - & 1)\left(n-2\right)c_{0}^{2}c_{3}){\left(r_{+}^{2}\right)}^{-1}+\left(\left(n-1\right)\left(n-2\right)\left(n-3\right)c_{0}^{3}c_{4}\right){\left(r_{+}^{3}\right)}^{-1}), \end{array}$$

where $r_{+}$ is the horizon radius. The thermodynamics of BH consists of many important thermodynamical quantities; entropy is one of the major thermodynamical quantities, which is defined as follows:

(5) $$S=\frac{r_{+}^{n-1}}{4}.$$

The Hawking temperature of BH can be obtained by using $T=\frac{\kappa }{2\pi }=\frac{1}{4\pi }\left(\frac{\partial \;\psi (r)}{\partial r}\right)$. The temperature of BH can also be obtained by using following relation [47]:

$$\begin{array}{c} T={\left(\frac{\partial M}{\partial S}\right)}_{Q}=\frac{{\left(\frac{\partial M}{\partial r_{+}}\right)}_{Q}}{{\left(\frac{\partial S}{\partial r_{+}}\right)}_{Q}}. \end{array}$$

By using the above relation, Hawking temperature of logarithmic charged BH turns out to be as follows:

(6) $$\begin{array}{ccc} T & = & \frac{(n-2)k}{4\pi r_{+}}-\frac{r_{+}\Lambda }{2\pi (n-1)}+\frac{m^{2}c_{0}}{4\pi r_{+}^{3}}\;ℸ+\frac{2\beta^{2}r_{+}}{\pi (n-1)}ℶ, \end{array}$$

where we use $ℸ=(c_{1}r_{+}^{3}+\left(n-2\right)c_{0}c_{2}r_{+}^{2}+\left(n-3\right)\left(n-2\right)c_{0}^{2}c_{3}r_{+}+\left(n-4\right)\left(n-3\right)\left(n-2\right)c_{0}^{3}c_{4})$ and $ℶ=\left(\ln\left(\frac{1+\sqrt{1+\frac{q^{2}}{\beta^{2}}r_{+}^{2-2n}})}{2}\right)+\left(1-\sqrt{1+\frac{q^{2}}{\beta^{2}}r_{+}^{2-2n}}\right))\right)$. The usual relation for BH entropy is $S_{o}=A/4$, where *A* is the area of the BH event horizon in the absence of the correction terms. The quantum corrections of gravity near the Planck scale changes the manifold structure of spacetime. This modifies the holographic principle, which leads to change in the entropy–area law of BH. The corrected entropy–area relation is given by the following [51,52,53]:

(7) $$\begin{array}{c} S=S_{o}-\frac{\xi }{2}\log\left(S_{o}T^{2}\right). \end{array}$$

By using Hawking temperature T and the entropy of zeroth order $S_{o}$ in the above relation, the corrected entropy of logarithmic charged BH turns out to be as follows:

(8) $$\begin{array}{c} S=\frac{r_{+}^{n-1}}{4}-\frac{\xi }{2}\log\left[\frac{r_{+}^{n-1}}{4}{\left(\frac{(n-2)k}{4\pi r_{+}}-\frac{r_{+}\Lambda }{2\pi }\left(n-1\right)+\frac{m^{2}c_{0}}{4\pi r_{+}^{3}}ℸ+\frac{2\;\beta^{2}r_{+}}{\pi (n-1)}ℶ\right)}^{2}\right]. \end{array}$$

**Figure 1:**
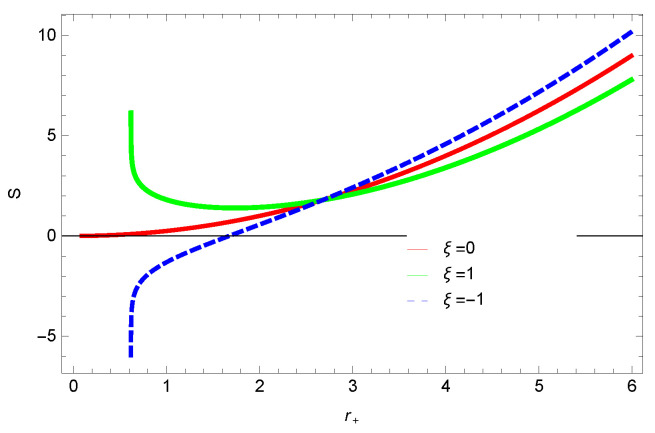
The corrected entropy of logarithmic charged BH in MG. We set β=1, n=3, $c_{0}=1$, $c_{1}=1$, $c_{2}=1$, $c_{3}=1$ and $c_{4}=1$.

**Figure 2:**
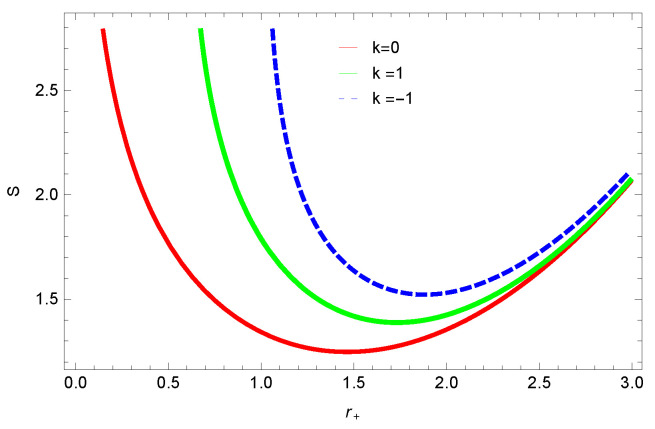
The corrected entropy of logarithmic charged BH in MG. We set β=1, n=3, $c_{0}=1$, $c_{1}=1$, $c_{2}=1$, $c_{3}=1$ and $c_{4}=1$.

**Figure 3:**
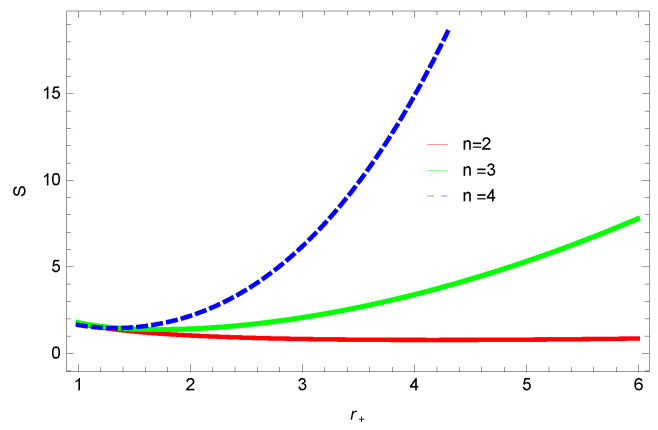
The corrected entropy of logarithmic charged BH in MG. We set β=1, $c_{0}=1$, $c_{1}=1$, $c_{2}=1$, $c_{3}=1$ and $c_{4}=1$.

**Figure 4:**
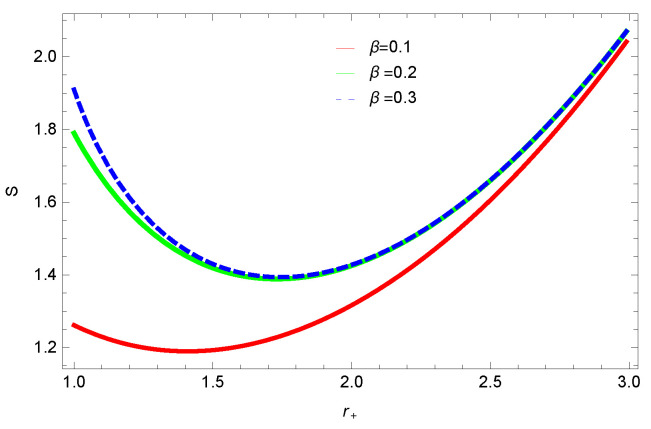
The corrected entropy of logarithmic charged BH in MG. We set n=3, $c_{0}=1$, $c_{1}=1$, $c_{2}=1$, $c_{3}=1$ and $c_{4}=1$.

Figure 1, Figure 2, Figure 3 and Figure 4 demonstrate the behavior of corrected entropy of logarithmic charged BH in MG vs $r_{+}$. These plots show the influence of corrected parameter ξ, topology of event horizon *k*, dimensions *n* and nonlinearity parameter β on the corrected entropy of BH. There exists a critical point in the corrected entropy of BH (Figure 1) at $r_{+}=2.6$. The behavior of the curves changes before and after the critical point, i.e., the corrected entropy increases for increasing values of the corrected parameter before the critical point, and it decreases after the critical point. For positive values of ξ, entropy increases, which represents stability. For negative ξ, entropy tends to decrease asymptotically, which shows the instability of BH; this behavior changes after the critical point. Figure 2 shows the behavior of corrected entropy for k=0 flat, k=1 spherical and k=−1 hyperbolic surfaces. We can observe that the corrected entropy increases for a hyperbolic surface, while it decreases for the case of a flat surface. Figure 3 and Figure 4 show the influence of different dimensions and the nonlinearity parameter on the corrected entropy of logarithmic charged BH in MG. For higher dimensions, the corrected entropy increases, which represents stability; for smaller dimension, the corrected entropy decreases. The impact of dimensions is significant for larger values of $r_{+}$. The corrected entropy increases with increasing values of the nonlinearity parameter for logarithmic charged BH in MG. For all the plots, as we increase $r_{+}$, the BH grows, and this causes the area of the event horizon to grow bigger, and hence, the entropy increases. For logarithmic charged BH in MG, we observe that the entropy increases exponentially.

The electrical charge per unit volume of BH is defined as follows [47]:

(9) $$\begin{array}{c} Q=\frac{q}{4\pi }. \end{array}$$

Another important thermodynamical quantity is volume $V={\left(\frac{\partial M}{\partial P}\right)}_{S,Q}$ of the BH which it is related to the mass and pressure of the BH. The relation for thermodynamic volume of the logarithmic charged BH becomes the following:

(10) $$\begin{array}{c} V=\frac{4}{3}\pi r_{+}^{3}. \end{array}$$

Helmholtz free energy (HFE) is the measure of useful work obtained from a closed thermodynamic system. The free energy *F* can be obtained from F=M−TS, and for the considered BH, it takes the following form:

(11) $$\begin{array}{ccc} F & = & kr_{+}^{n-2}+\frac{8\beta^{2}r_{+}^{n}}{n(n-1)}(\ln\frac{\left(1+\sqrt{1+\frac{q^{2}}{\beta^{2}}r^{2-2n}}\right)}{2}+(\left(2n-1\right)(1-(1+\frac{q^{2}}{\beta^{2}}\\ & \times & r^{2-2n}{)}^{\frac{1}{2}}{))\left(n\right)}^{-1})-\frac{2r_{+}^{n}\Lambda }{n(n-1)}+\frac{8\left(n-1\right)q^{2}r_{+}^{-2n}}{n^{2}\left(n-2\right)}F(\frac{1}{2},\frac{n-2}{2(n-1)},(4\\ & - & {3n)\left(2-2n\right)}^{-1},\frac{-q^{2}r_{+}^{2-2n}}{\beta^{2}})+\frac{c_{0}m^{2}r_{+}^{n-1}}{n-1}(c_{1}+\frac{\left(n-1\right)c_{0}c_{2}}{r_{+}}+((n\\ & - & 1)\left(n-2\right)c_{0}^{2}c_{3}){\left(r_{+}^{2}\right)}^{-1}+\left(\left(n-1\right)\left(n-2\right)\left(n-3\right)c_{0}^{3}c_{4}\right){\left(r_{+}^{3}\right)}^{-1})-(\\ & \times & \left(\left(n-2\right)k\right){\left(4\pi r_{+}\right)}^{-1}-\frac{r_{+}\Lambda }{2\pi (n-1)}+\frac{m^{2}c_{0}}{4\pi r_{+}^{3}}\;ℸ+\frac{2\beta^{2}r_{+}}{\pi (n-1)}ℶ)\Delta , \end{array}$$

where in the above relation, $\Delta =\frac{r_{+}^{n-1}}{4}-\frac{\xi }{2}\log\left[\frac{r_{+}^{n-1}}{4}{\left(\frac{(n-2)k}{4\pi r_{+}}-\frac{r_{+}\Lambda }{2\pi }\left(n-1\right)+\frac{m^{2}c_{0}}{4\pi r_{+}^{3}}+\frac{2\;\beta^{2}r_{+}}{\pi (n-1)}ℶ\right)}^{2}\right]$.

**Figure 5:**
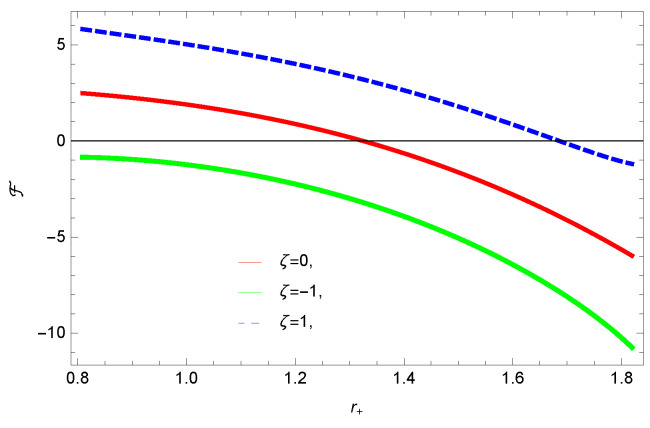
The HFE of logarithmic charged BH in MG. We set n=3, $c_{0}=1$, $c_{1}=1$, $c_{2}=1$, $c_{3}=1$ and $c_{4}=1$.

**Figure 6:**
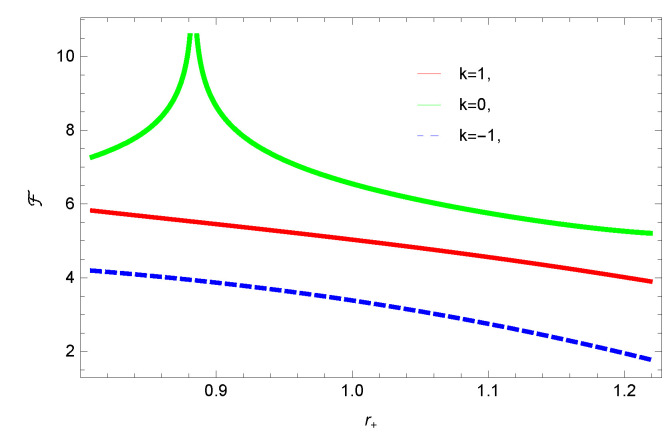
The HFE of logarithmic charged BH in MG. We set n=3, $c_{0}=1$, $c_{1}=1$, $c_{2}=1$, $c_{3}=1$ and $c_{4}=1$.

**Figure 7:**
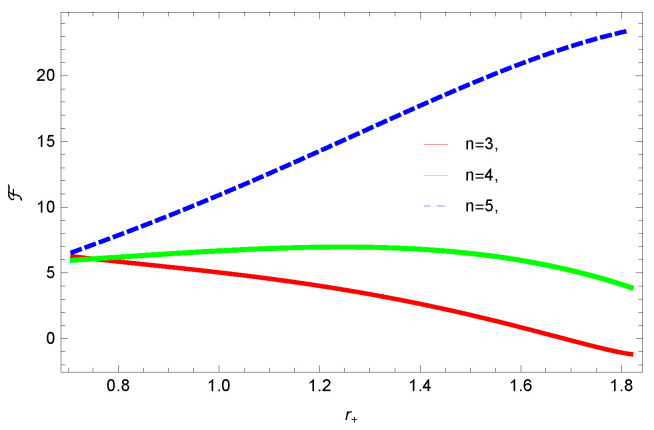
The HFE of logarithmic charged BH in MG. We set β=1, $c_{0}=1$, $c_{1}=1$, $c_{2}=1$, $c_{3}=1$ and $c_{4}=1$.

**Figure 8:**
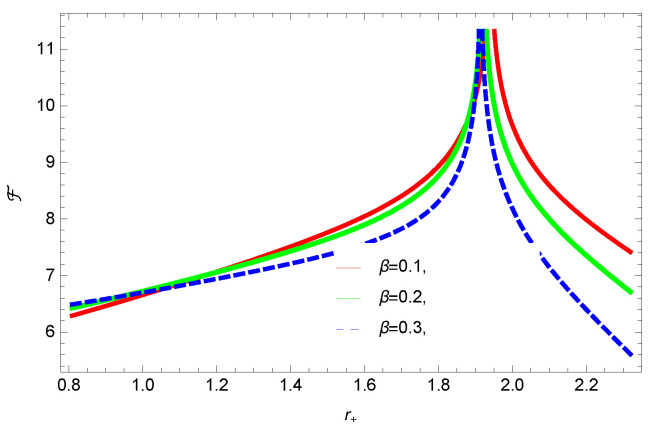
The HFE of logarithmic charged BH in MG. We set n=3, $c_{0}=1$, $c_{1}=1$, $c_{2}=1$, $c_{3}=1$ and $c_{4}=1$.

Figure 5, Figure 6, Figure 7 and Figure 8 demonstrate the corrected HFE behavior of logarithmic charged BH in massive gravity vs. $r_{+}$. These plots show the influence of corrected parameter ξ, topology of event horizon *k*, dimensions *n* and nonlinearity parameter β on the free energy of BH. From Figure 5, one can clearly see that the positive values of ξ increase the HFE, which indicates the instability. In contrast, negative ξ decreases HFE. This plot helps us to identify the region of instability. A negative value of Helmholtz free energy will not be able to extract any useful work from the concerned BH. For ξ=−1, *F* changes the sign from positive to negative, which represents a phase transition. Figure 6 shows the behavior of HFE for k=0 flat, k=1 spherical and k=−1 hyperbolic surfaces. We can observe that there exists discontinuity in the plot for the case of the flat surface. The strange plot of HFE is due to the overcome of quantum effects by tidal forces at very small values of $r_{+}$. Figure 7 and Figure 8 show the influence of different dimensions and nonlinearity parameters on the HFE of logarithmic charged BH in MG. HFE increases with increasing dimensions, while it decreases with increasing values of the nonlinearity parameter. The HFE decreases w.r.t $r_{+}$, and there exists discontinuity, which is due to the overcome of quantum effects by tidal forces.

By using $\varphi ={\left(\frac{\partial M}{\partial Q}\right)}_{S}=\frac{{\left(\frac{\partial M}{\partial q}\right)}_{r_{+}}}{{\left(\frac{\partial Q}{\partial q}\right)}_{r_{+}}}$ the relation for chemical potential of the considered BH turns out to be the following:

(12) $$\begin{array}{ccc} \varphi & = & 4\pi (\frac{16\left(n-1\right)q\;r_{+}^{2-n}}{n^{2}\left(n-2\right)}F\left(\frac{1}{2},\frac{n-2}{2(n-1)},\frac{4-3n}{2-2n},\frac{-q^{2}\;r_{+}^{2-2n}}{\beta^{2}}\right)-(16(n\\ & - & 1)q\;r_{+}^{2-n}){\left(n^{2}\left(n-2\right)\beta^{2}\right)}^{-1}F^{1}\left(\frac{1}{2},\frac{n-2}{2(n-1)},\frac{4-3n}{2-2n},\frac{-q^{2}r_{+}^{2-2n}}{\beta^{2}}\right)+(8r_{+}^{n}\\ & \times & \left(\frac{-\left(-1+2n\right)q^{2}r^{1-2n}}{2\sqrt{1+\frac{q^{2}r^{2-2n}}{\beta^{2}}}\beta^{2}}+\frac{\ln\left(2-2n\right)q^{2}r^{1-2n}}{4\sqrt{1+\frac{q^{2}r^{2-2n}}{\beta^{2}}}\beta^{2}}\right)\beta^{2})n(n-1)). \end{array}$$

Moreover, the internal energy for the logarithmic charged BH can be obtained by using E=F+TS. It takes the following form

(13) $$\begin{array}{ccc} E & = & kr_{+}^{n-2}-\frac{2r_{+}^{n}\Lambda }{n(n-1)}+\frac{8\beta^{2}r_{+}^{n}}{n(n-1)}ℶ+\frac{\left(2n-1\right)\left(1-\sqrt{1+\frac{q^{2}}{\beta^{2}}r_{+}^{2-2n}}\right)}{n})\\ & + & \frac{8\left(n-1\right)q^{2}r_{+}^{-2n}}{n^{2}\left(n-2\right)}F\left(\frac{1}{2},\frac{n-2}{2(n-1)},\frac{4-3n}{2-2n},\frac{-q^{2}r_{+}^{2-2n}}{\beta^{2}}\right)+\frac{c_{0}m^{2}r_{+}^{n-1}}{n-1}(c_{1}\\ & + & \frac{\left(n-1\right)c_{0}c_{2}}{r_{+}}+\frac{\left(n-1\right)\left(n-2\right)c_{0}^{2}c_{3}}{r_{+}^{2}}+\frac{\left(n-1\right)\left(n-2\right)\left(n-3\right)c_{0}^{3}c_{4}}{r_{+}^{3}})\\ & - & \frac{(n-2)k}{4\pi r_{+}}-\left(\frac{r_{+}\Lambda }{2\pi (n-1)}+\frac{m^{2}c_{0}}{4\pi r_{+}^{3}}\;ℸ+\frac{2\beta^{2}r_{+}}{\pi (n-1)}ℶ\right)\Delta +\frac{(n-2)k}{4\pi r_{+}}\\ & - & \left(\frac{r_{+}\Lambda }{2\pi (n-1)}+\frac{m^{2}c_{0}}{4\pi r_{+}^{3}}\;ℸ+\frac{2\beta^{2}r_{+}}{\pi (n-1)}ℶ\right)\Delta . \end{array}$$

**Figure 9:**
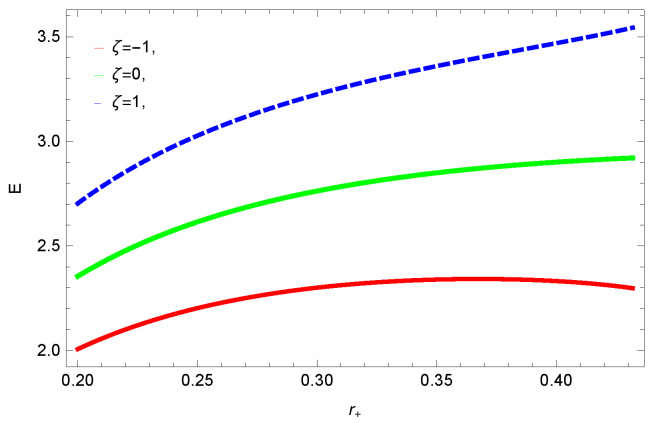
The internal energy of logarithmic charged BH in MG. We set n=3, $c_{0}=1$, $c_{1}=1$, $c_{2}=1$, $c_{3}=1$ and $c_{4}=1$.

**Figure 10:**
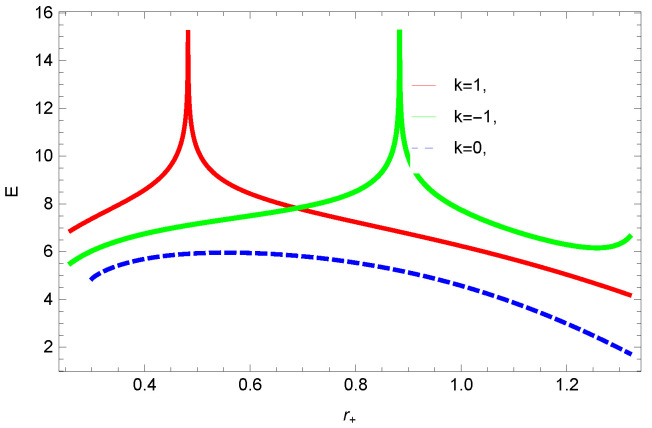
The internal energy of logarithmic charged BH in MG. We set n=3, $c_{0}=1$, $c_{1}=1$, $c_{2}=1$, $c_{3}=1$ and $c_{4}=1$.

**Figure 11:**
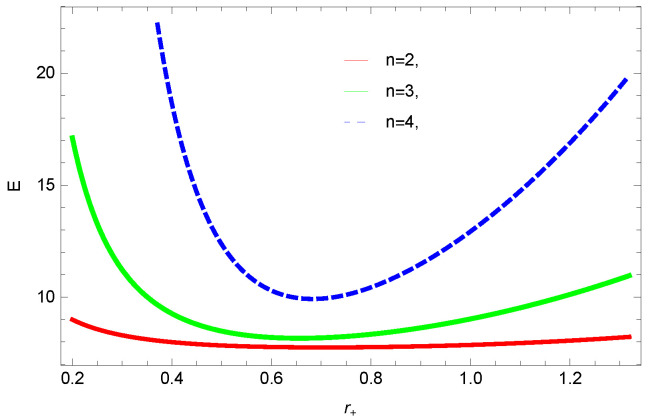
The internal energy of logarithmic charged BH in MG. We set β=3, $c_{0}=1$, $c_{1}=1$, $c_{2}=1$, $c_{3}=1$ and $c_{4}=1$.

**Figure 12:**
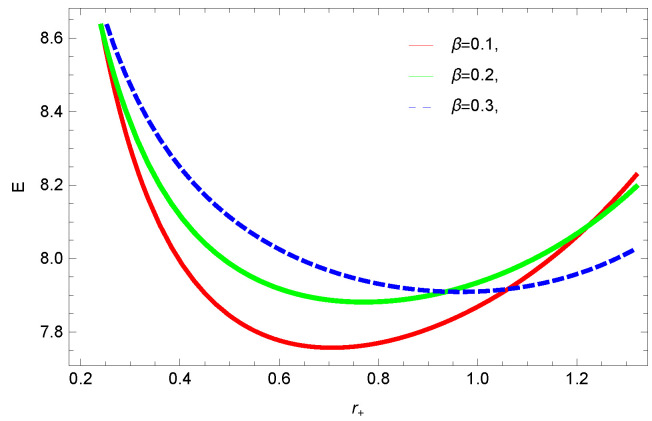
The internal energy of logarithmic charged BH in MG. We set n=3, $c_{0}=1$, $c_{1}=1$, $c_{2}=1$, $c_{3}=1$ and $c_{4}=1$.

Figure 9, Figure 10, Figure 11 and Figure 12 demonstrate the internal energy behavior of logarithmic charged BH in MG vs. $r_{+}$. For the negative value of ξ, the internal energy decreases, and it increases for the positive value. This is in agreement with the first law of BH thermodynamics, as we have already observed that entropy increases for negative value of ξ. Thus, even after incorporating the quantum correction, the first laws of BH thermodynamics hold. The decrease in internal energy of logarithmic charged BH in massive gravity is due to the quantum corrections, which lead to stability of the BHs. For k=1 spherical and k=−1 hyperbolic surface, the behavior of internal energy of the considered BH is quite different; this different behavior is due to the quantum corrections in the internal energy. The internal energy increases with increasing the dimension and nonlinearity parameter of BH.

The modified pressure can be calculated by using $P=-(\frac{dF}{dV})$; see Appendix A.

**Figure 13:**
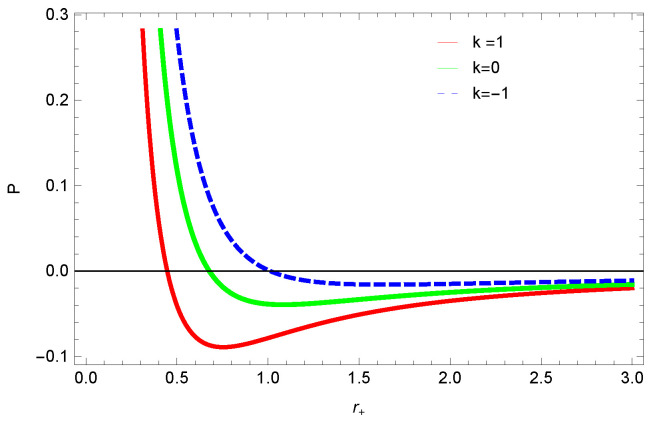
The corrected pressure of logarithmic charged BH in MG. We set $c_{0}=1$, β=1, $c_{1}=1$, n=3, $c_{2}=1$, $c_{3}=1$ and $c_{4}=1$.

**Figure 14:**
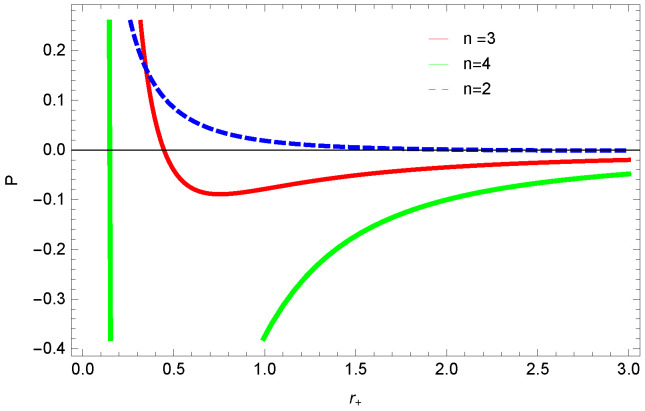
The corrected pressure of logarithmic charged BH in MG. We set β=1, $c_{0}=1$, $c_{1}=1$, β=3, $c_{3}=1$, $c_{2}=1$ and $c_{4}=1$.

**Figure 15:**
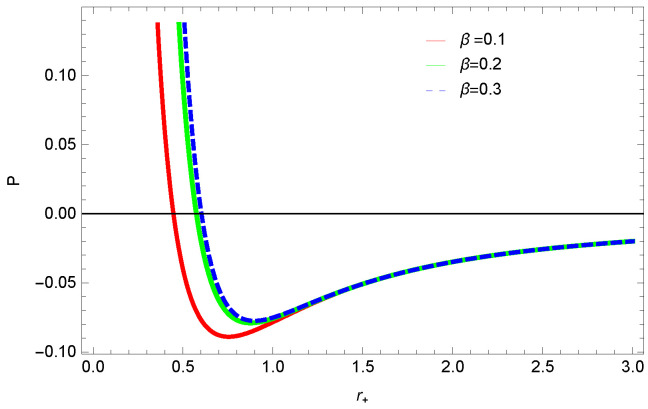
The corrected pressure of logarithmic charged BH in MG. We set n=3, $c_{0}=1$, $c_{1}=1$, $c_{2}=1$, $c_{3}=1$ and $c_{4}=1$.

Figure 13, Figure 14 and Figure 15 demonstrate the corrected pressure behavior of logarithmic charged BH in MG vs. $r_{+}$. One can observe that for positive values of *k*, the pressure becomes negative and it shifts toward a positive value for negative *k*. This justifies that negative *k* increases the stability of the BH, which follows the previous cases of entropy, internal energy and free energy. The impact of different dimensions *n* is quite interesting on the corrected pressure. The pressure remains positive throughout the range of $r_{+}$ for the lower dimension of *n*, and it becomes negative; there exists discontinuity for higher dimensions. The influence of the nonlinearity parameter is significant only for smaller BHs, and pressure increases with increasing values of the nonlinearity parameter.

The enthalpy is another significant thermodynamical quantity, which measures the energy changes of the system. It also helps us to determine the equilibrium conditions of the system. The enthalpy in BH thermodynamics has become more significant after its inclusion in first law of BH thermodynamics. The mass *M* of an AdS BH works as enthalpy in classical thermodynamics. The enthalpy can be calculated by using H=E+PV; see Appendix B for mathematical relation.

**Figure 16:**
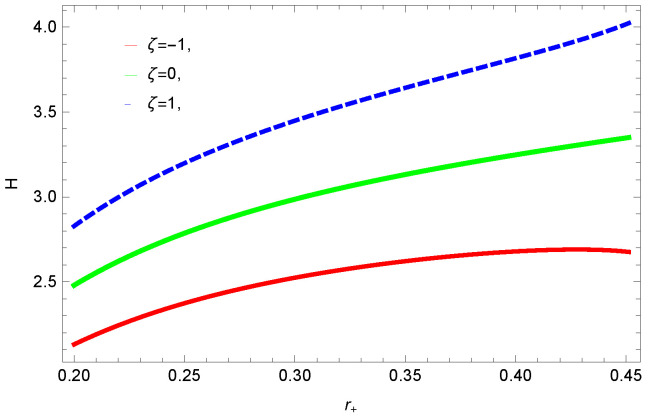
The enthalpy of logarithmic charged BH in MG. We set n=3, $c_{0}=1$, β=1, $c_{2}=1$, $c_{3}=1$, $c_{1}=1$, and $c_{4}=1$.

**Figure 17:**
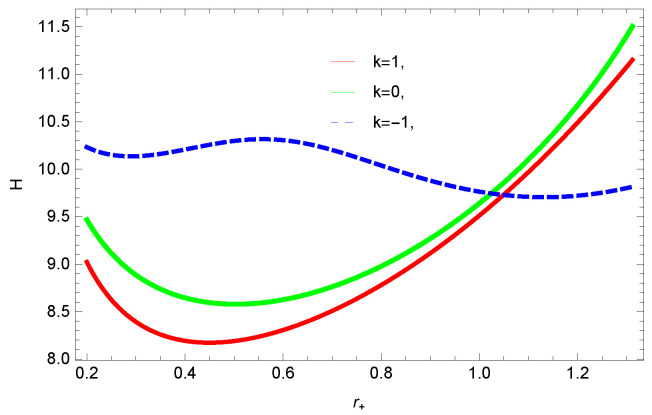
The enthalpy of logarithmic charged BH in MG. We set $c_{0}=1$, $c_{1}=1$, β=1, n=3, $c_{3}=1$, $c_{2}=1$, and $c_{4}=1$.

**Figure 18:**
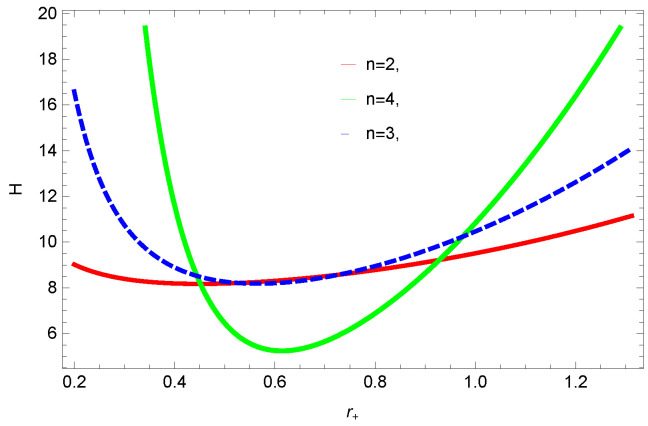
The enthalpy of logarithmic charged BH in MG. We set k=1, $c_{0}=1$, β=1, $c_{2}=1$, $c_{1}=1$, $c_{3}=1$ and $c_{4}=1$.

**Figure 19:**
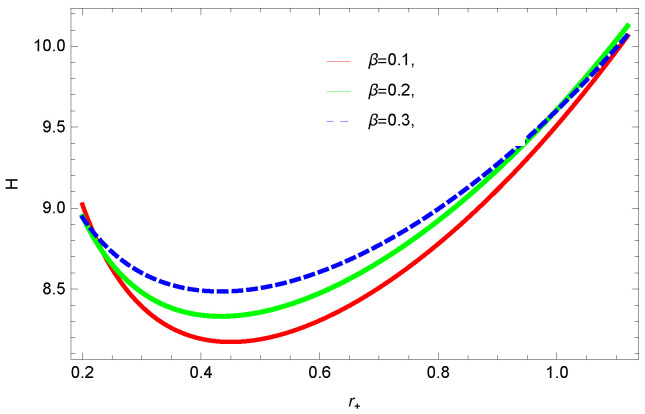
The enthalpy of logarithmic charged BH in MG. We set k=1, $c_{0}=1$, $c_{1}=1$, n=3, $c_{3}=1$, $c_{2}=1$, and $c_{4}=1$.

Figure 16, Figure 17, Figure 18 and Figure 19 demonstrate the enthalpy behavior of logarithmic charged BH in MG vs. $r_{+}$. From the plots, it is clear that enthalpy increases with increasing the value of $r_{+}$. The negative correction parameter decreases the enthalpy and hence, induces stability; there exist discontinuities in the plots of enthalpy for spherical and hyperbolic surfaces, which is because of the overcome of quantum effects by tidal forces. The enthalpy of logarithmic charged BH in MG increases with increasing dimensions and the nonlinearity parameter.

In order to have the static boundary of BH at a fixed temperature, one should have fixed pressure and temperature. In this scenario, the thermodynamical potential to be utilized is the Gibbs free energy. Once we have relations for HFE, pressure and volume, it is simple to calculate the Gibbs free energy by using G=F+PV; see Appendix C.

**Figure 20:**
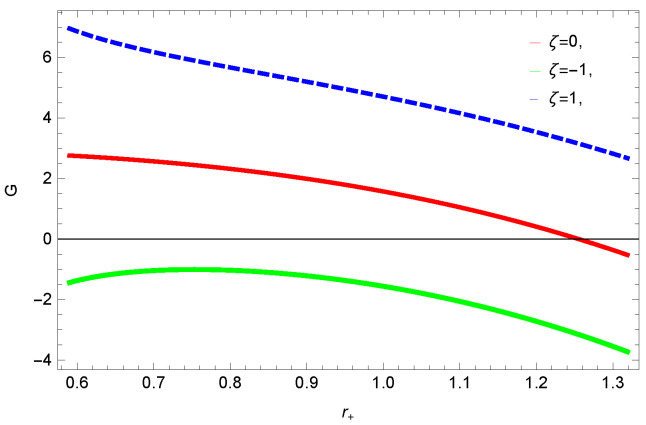
The Gibbs free energy of logarithmic charged BH in MG. We set $c_{0}=1$, $c_{1}=1$, k=1, $c_{2}=1$, n=3, $c_{3}=1$ and $c_{4}=1$.

**Figure 21:**
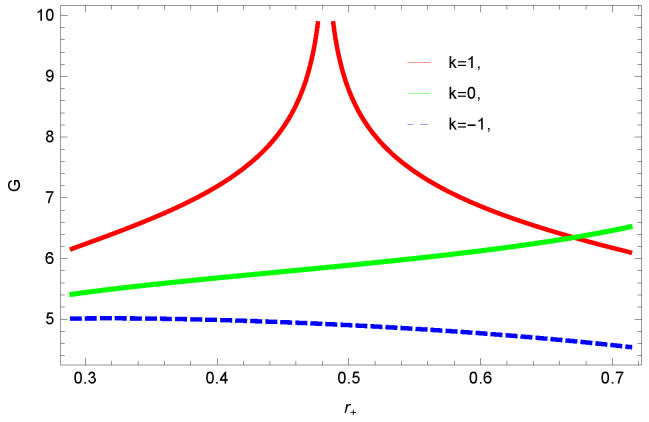
The Gibbs free energy of logarithmic charged BH in MG. We set n=3, $c_{0}=1$, β=1, $c_{2}=1$, $c_{1}=1$, $c_{3}=1$ and $c_{4}=1$.

**Figure 22:**
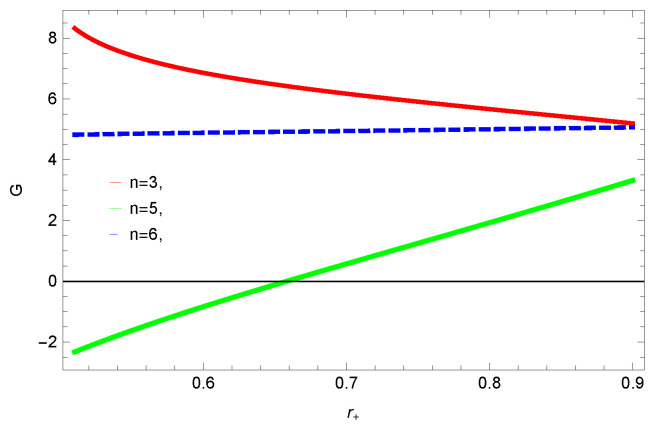
The Gibbs free energy of logarithmic charged BH in MG. We set k=1, β=1, $c_{0}=1$, $c_{1}=1$, $c_{2}=1$, $c_{3}=1$ and $c_{4}=1$.

**Figure 23:**
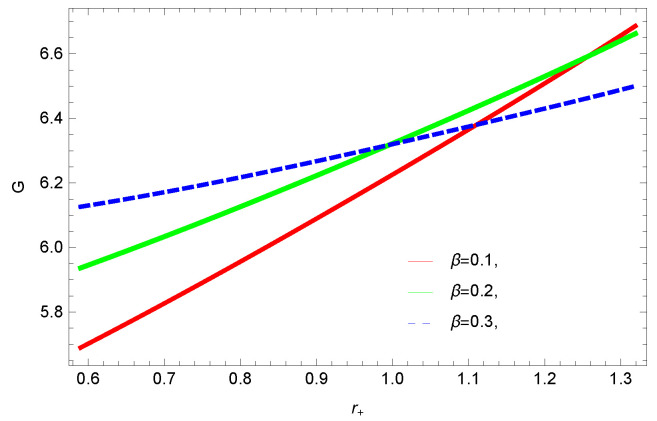
The Gibbs free energy of logarithmic charged BH in MG. We set k=1, n=3, $c_{0}=1$, $c_{1}=1$, $c_{2}=1$, $c_{3}=1$ and $c_{4}=1$.

To study the impact of parameters on the global stability of BH, we plot the relation of Gibbs free energy in Figure 20, Figure 21, Figure 22 and Figure 23. G>0 represents the global stability of the BH, while G<0 shows the region of global instability. The plots show that for negative correction parameter ξ=−1, the Gibbs free remains negative throughout the horizon radius, which represents the global instability of BH. For ξ=0,1, smaller BHs remain stable while larger BHs become instable. The Gibbs free energy remains positive for k=0 flat, k=1 spherical and k=−1 hyperbolic surfaces, which is a sign of global stability. There exists discontinuity in the plot for spherical surface, which represents the global instability; this unusual behavior is due to the overcome of quantum effects by tidal forces.

## 3. Holographic Duality and Thermal Stability

The van der Waals system is one of the most relevant models for discussing the liquid–gas system and its critical characteristics. The modification in ideal gas equation provides the equation of state for this model. The van der Waals model is given as follows [54]:

(14) $$\begin{array}{c} T(P,V)k=\left(P_{v}+\frac{a}{V^{2}}\right)\left(V-B\right), \end{array}$$

where *P*, *T* and *V* are pressure, temperature and specific volume, respectively, *B* and *a* represent the size and strength of attraction of the molecules. Here, k=1 is the Boltzmann constant. One can obtain the ideal gas law by setting a=B=0. The relationship between the van der Waals and BH helps us to consider the analogy between the temperature of the fluid and the temperature of the BH. The above model can also be expressed as follows:

(15) $$\begin{array}{c} P_{v}=\frac{T}{\left(V-B\right)}-\frac{a}{V^{2}}. \end{array}$$

In order to have holographic duality of charged BH in MG, the condition $P=P_{v}$ must hold. By using the temperature and thermodynamic volume of BH, one can obtain the pressure as follows:

(16) $$\begin{array}{c} P_{v}=\frac{3}{4\pi r_{+}^{3}}\left(\frac{(n-2)k}{4\pi r_{+}}-\frac{r_{+}\Lambda }{2\pi (n-1)}+\frac{m^{2}c_{0}}{4\pi r_{+}^{3}}\;ℸ+\frac{2\beta^{2}r_{+}}{\pi (n-1)}ℶ\right). \end{array}$$

**Figure 24:**
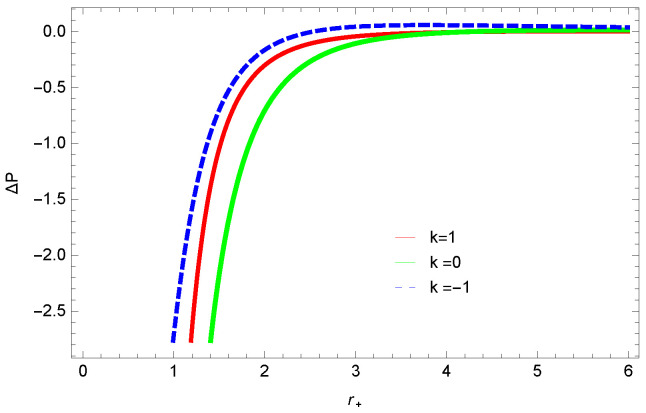
The plot of $\Delta P=P-P_{v}$ in terms of $r_{+}$. We set n=3, $c_{0}=1$, $c_{1}=1$, $c_{2}=1$, $c_{3}=1$ and $c_{4}=1$.

**Figure 25:**
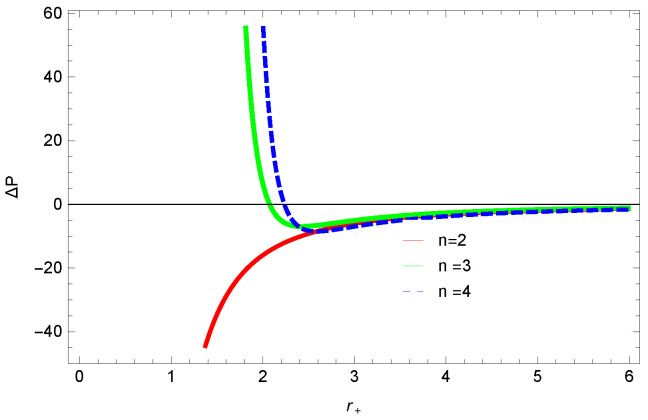
The plot of $\Delta P=P-P_{v}$ in terms of $r_{+}$. We set k=1, $c_{0}=1$, $c_{1}=1$, $c_{2}=1$, $c_{3}=1$ and $c_{4}=1$.

**Figure 26:**
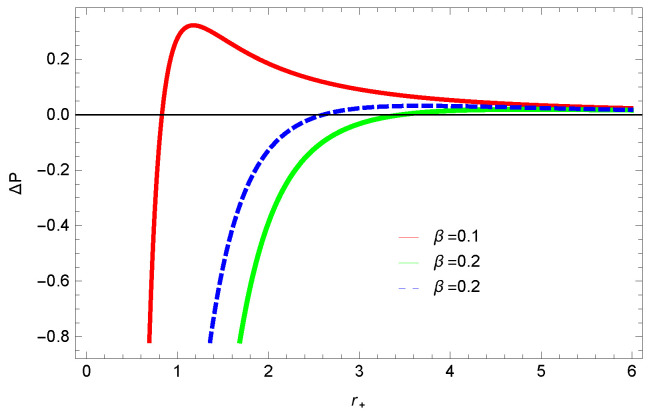
The plot of $\Delta P=P-P_{v}$ in terms of $r_{+}$. We set n=3, $c_{0}=1$, $c_{1}=1$, $c_{2}=1$, $c_{3}=1$ and $c_{4}=1$.

Figure 24, Figure 25 and Figure 26 demonstrate the behavior $\Delta P=P-P_{v}$ in terms of V. For large BHs, ΔP→0, which shows that the thermal correction has no significant role to disturb the holographic duality of the logarithmic charged BH in MG. The influence of the topology of the event horizon *k* and nonlinearity parameter is opposite.

The stability of BH can be considered in both canonical and grand canonical ensembles. In canonical ensemble, C>0 along with T>0 represents the thermal stability, while C<0 represents the unstable region, and the phase transition occurs in an unstable region to attain stability. The specific heat is defined as follows [47]:

(17) $$\begin{array}{c} C=T\left(\frac{\partial S}{\partial T}\right)=T\left(\frac{\partial S}{\partial r_{+}}\right){\left(\frac{\partial T}{\partial r_{+}}\right)}^{-1}, \end{array}$$

Now, by plugging the relations of entropy and temperature of the logarithmic charged BH in MG in the above equation, we can obtain the specific heat (see Appendix D).

**Figure 27:**
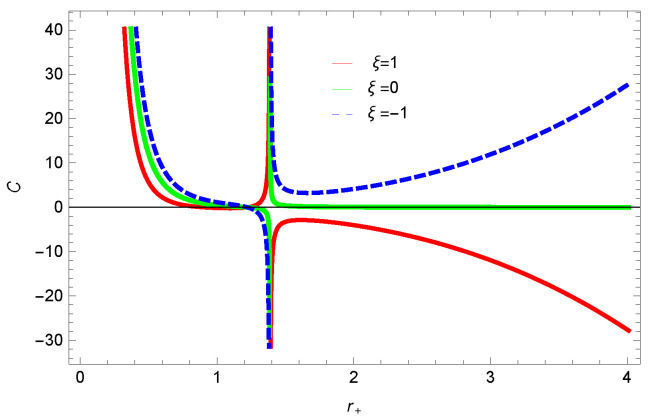
Specific heat of logarithmic charged BH in MG. We set k=1, n=3, $c_{0}=1$, $c_{1}=1$, $c_{2}=1$, $c_{3}=1$ and $c_{4}=1$.

**Figure 28:**
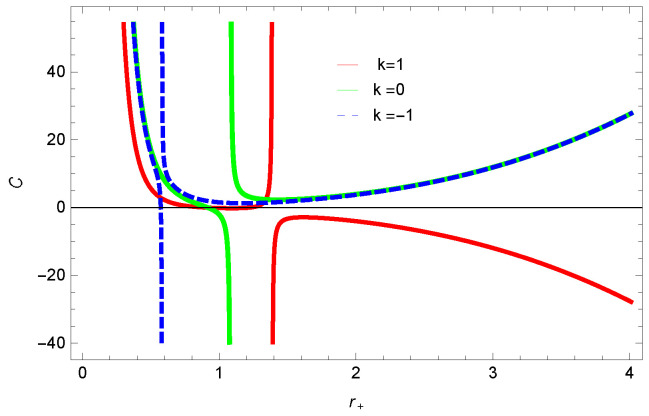
Specific heat of logarithmic charged BH in MG. We set β=1, n=3, $c_{0}=1$, $c_{1}=1$, $c_{2}=1$, $c_{3}=1$ and $c_{4}=1$.

**Figure 29:**
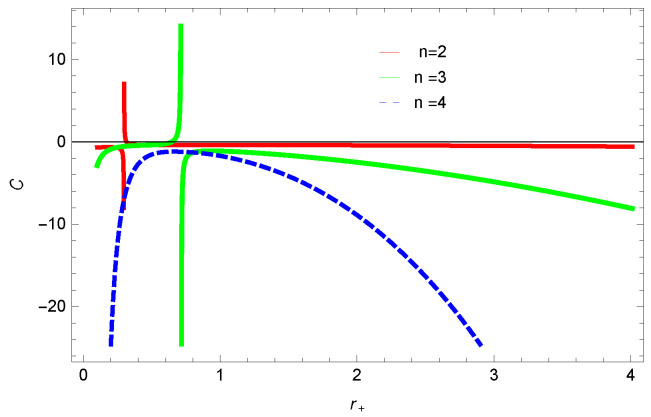
Specific heat of logarithmic charged BH in MG. We set β=1, k=1, $c_{0}=1$, $c_{1}=1$, $c_{2}=1$, $c_{3}=1$ and $c_{4}=1$.

**Figure 30:**
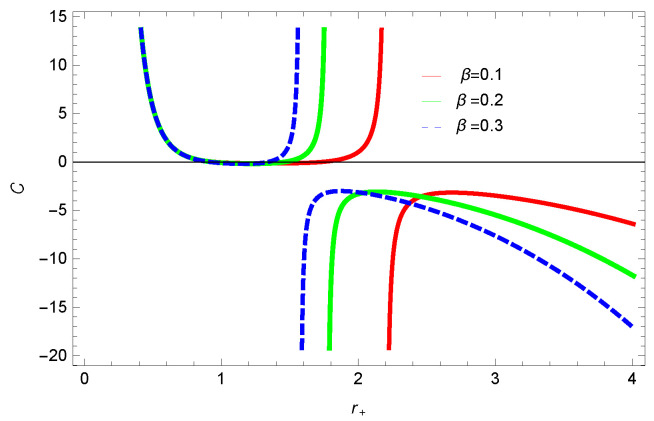
Specific heat of logarithmic charged BH in MG. We set n=2, k=1, $c_{0}=1$, $c_{1}=1$, $c_{2}=1$, $c_{3}=1$ and $c_{4}=1$.

For unstable BH, phase transition occurs to gain stability. The roots and points of divergences of *C* provides the phase transition points. The roots of the heat capacity help us to determine the thermal transitions between the physical and un-physical states of BH. For the considered BH, as the roots and divergences of the heat capacity are not possible analytically, we have shown the behavior specific heat of logarithmic charged BH in MG in Figure 27, Figure 28, Figure 29 and Figure 30. One can see that in the plot of Figure 27, the divergence in the heat capacity occurs at $r_{+}=1.4$. The heat capacity remains positive before and after the point of divergence for ξ=0,−1, which represent the stable phase. The phase transition occurs at the point of divergence, and the negative range of *C* shows the region of instability of BH. There exist discontinuities or divergences for k=0 flat, k=1 spherical and k=−1 hyperbolic surfaces for smaller BHs—which represents instability, while for larger values of $r_{+}$, BHs becomes stable for flat and hyperbolic surfaces. The nonlinearity parameter β shows the significant role on the specific heat of the considered BH. For the small BH, the positive heat capacity shows the local stability, while for large BH, C<0 and discontinuity in heat capacity represent the local instability. One can see that for logarithmic charged BH in higher dimensions, the instability of BH increases.

The determinant of the Hessian matrix *H* helps us to discuss the local stability. As the charge remains as a fixed parameter in the canonical ensemble, the positive region of heat capacity *C* is sufficient for the stability of BH. However, we need to examine the sign of *H* to examine the thermal stability in the grand canonical ensemble. The Hessian matrix is defined as follows [47]:

(18) $$\begin{array}{c} H_{X_{i},X_{j}}^{F}=\frac{\partial^{2}F}{\partial X_{i}\partial X_{j}}=\;\left[\begin{array}{cc} \frac{\partial^{2}F}{\partial T^{2}} & \frac{\partial^{2}F}{\partial T\;\partial \eta }\\ \frac{\partial^{2}F}{\partial \eta \;\partial T} & \frac{\partial^{2}F}{\partial \eta^{2}} \end{array}\right] \end{array}$$

From the above equation, by setting $H_{X_{i},X_{j}}^{F}=0$, one can easily obtain the following relation:

(19) $$\begin{array}{c} \left(\frac{\partial^{2}F}{\partial T^{2}}\right)\left(\frac{\partial^{2}F}{\partial \eta^{2}}\right)=\left(\frac{\partial^{2}F}{\partial T\partial \eta }\right)\left(\frac{\partial^{2}F}{\partial \eta \partial T}\right) \end{array}$$

(20) $$\begin{array}{c} \tau =T\tau \left(H\right)=\tau_{1}+\tau_{2} \end{array}$$

where $\tau_{1}=\left(\frac{\partial^{2}F}{\partial T^{2}}\right)$ and $\tau_{2}=\left(\frac{\partial^{2}F}{\partial \eta^{2}}\right)$. The important condition for the stability of BH is τ≥0.

**Figure 31:**
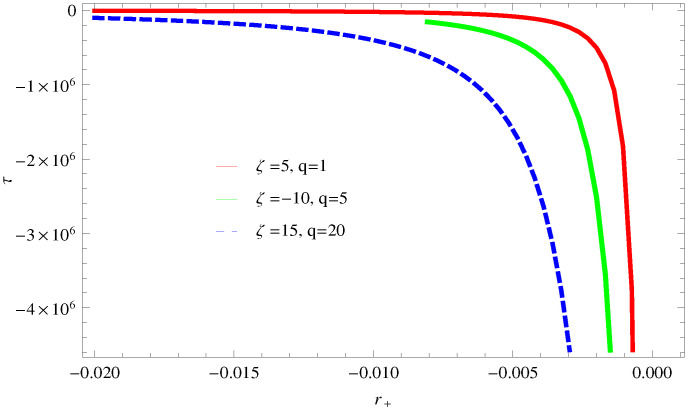
Plot of τ versus horizon radius $r_{+}$ for different values of ξ.

Figure 31 demonstrates the behavior of trace of Hessian matrix τ verses $r_{+}$ for different values of ξ. The necessary condition for the stability is τ≥0; one can observe this for all the values of the corrected parameters ξ, τ≤0, which show the local instability of logarithmic charged BH in the MG.

## 4. Concluding Remarks

In this work, we considered the logarithmic charged BH in MG with the negative cosmological constant and studied the corrected thermodynamics. We computed the relations of logarithmic corrected *S*, *F*, *E*, *H*, *G* and *C*. In order to investigate the local and global stability and phase transition points, we plotted these relations against horizon radius $r_{+}$. We provided the deep analysis of the impact of corrected parameter ξ, topology of event horizon *k*, dimensions *n* and nonlinearity parameter β on the thermodynamical quantities of the BH.

We found that there exists a critical point in the corrected entropy of BH (Figure 6) at $r_{+}=2.6$, and the behavior of the curve changes before and after the critical point. The corrected entropy increases for increasing values of corrected parameter ξ before the critical point, and it decreases after the critical point. For positive values of ξ, the entropy increases, which represents the stability, while for negative ξ, the entropy tends to decrease asymptotically, which shows the instability of BH. This behavior changes after the critical point. The HFE helped us to identify the region of instability for ξ=−1, as *F* changes the sign from positive to negative, which represents a phase transition. For negative values of ξ, the internal energy decreases, while it increases for positive values, which is in agreement with the first law of BH thermodynamics. The decrease in internal energy of logarithmic charged BH in MG is due to the quantum corrections, which lead to stability of the BHs. We also observed that for the negative correction parameter ξ=−1, the Gibbs free remains negative throughout the horizon radius, which represents the global instability of BH. For ξ=0,1 smaller BHs remain stable, while larger BHs become unstable. The Gibbs free energy remains positive for k=0 flat, k=1 spherical and k=−1 hyperbolic surfaces, which is sign of global stability. We analyzed that in the plot of Figure 27, the divergence in the heat capacity occurred at $r_{+}=1.4$. The heat capacity remained positive before and after the point of divergence for ξ=0,−1, which showed the stable phase. Phase transition occurred at the point of divergence, and the negative range of *C* showed the region of instability of BH. For large BHs, ΔP→0, which showed that thermal correction has no significant role in disturbing the holographic duality of the logarithmic charged BH in MG.

## Data Availability

All the related data is included in the manuscript.

## Appendix A

For the logarithmic charged BH in MG, the modified pressure becomes:

$$\begin{array}{ccc} P & = & -4\pi r_{+}^{2}(\frac{k(n-2)}{4\pi r_{+}^{2}}+k\left(n-2\right)r_{+}^{n-3}-\frac{\Lambda }{2\pi (n-1)}-\frac{2r_{+}^{n-1}\Lambda }{\left(n-1\right)}+(8r^{n-1}\\ & \times & \beta^{2}(\left(\left(2n-1\right)\left(1-\sqrt{1+\frac{q^{2}r^{2-2n}}{\beta^{2}}}\right)\right){\left(n\right)}^{-1}+\frac{1}{2}\ln(1+\sqrt{1+\frac{q^{2}r^{2-2n}}{\beta^{2}}}\\ & \times & {)))\left(n-1\right)}^{-1}+\frac{8r^{n}\beta^{2}\left(\frac{ln\left(2n-n\right)q^{2}r^{1-2n}}{4\beta^{2}\left(1+\sqrt{1+\frac{q^{2}r^{2-2n}}{\beta^{2}}}\right)}-\frac{\left(2-2n\right)\left(2n-1\right)q^{2}r^{1-2n}}{2n\left(1+\sqrt{1+\frac{q^{2}r^{2-2n}}{\beta^{2}}}\right)\beta^{2}}\right)}{n(n-1)}+(8\\ & \times & \left(2-n\right)\left(n-1\right)q^{2}r^{1-n}F\left(\frac{1}{2},\frac{n-2}{2(n-1)},\frac{4-3n}{2-2n},\frac{-q^{2}r_{+}^{2-2n}}{\beta^{2}}\right))(n^{2}\left(n-2\right)\\ & \times & {)}^{-1}-(8\left(2-2n\right)\left(-1+n\right)q^{4}r^{3-3n}\;F^{1}(\frac{1}{2},\frac{n-2}{2(n-1)},\frac{4-3n}{2-2n},\frac{-q^{2}r_{+}^{2-2n}}{\beta^{2}}\\ & \times & )){\left(\left(n-2\right)n^{2}\beta^{2}\right)}^{-1}+\frac{c_{0}m^{2}r_{+}^{n-1}}{\left(n-1\right)}(-\frac{\left(n-1\right)c_{0}c_{2}}{r_{+}^{2}}-(2\left(n-1\right)\left(n-2\right)c_{0}^{2}\\ & \times & c_{3}){\left(r_{+}^{3}\right)}^{-1}-\left(3\left(n-1\right)\left(n-2\right)\left(n-3\right)c_{0}^{3}c_{4}\right){\left(r_{+}^{4}\right)}^{-1})+c_{0}m^{2}r_{+}^{n-2}(c_{1}+(\\ & \times & \left(n-1\right)c_{0}c_{2}){\left(r_{+}\right)}^{-1}+\left(\left(n-1\right)\left(n-2\right)c_{0}^{2}c_{3}\right){\left(r_{+}^{2}\right)}^{-1}+(\left(n-1\right)\left(n-2\right)(n\\ & - & 3)c_{0}^{3}c_{4}){\left(r_{+}^{3}\right)}^{-1})+\Delta (\frac{k(n-2)}{4\pi r_{+}^{2}}+\left(\Lambda \right){\left(2\pi \left(n-1\right)\right)}^{-1}-(m^{2}c_{0}(3r_{+}^{2}c_{1}+2\\ & \times & \left(n-2\right)rc_{0}c_{2}+\left(n-3\right)\left(n-2\right)c_{0}^{2}c_{3})){\left(4\pi r_{+}^{3}\right)}^{-1}+\frac{3m^{2}c_{0}}{4\pi r_{+}^{4}}ℸ-(2\beta^{2}(1+\frac{1}{2}\\ & \times & \ln\left(1+{\left(1+\left(q^{2}r^{2-2n}\right){\left(\beta^{2}\right)}^{-1})\right)}^{\frac{1}{2}}-{\left(1+\left(q^{2}r^{2-2n}\right)\beta^{2}\right)}^{\frac{1}{2}}))\right){\left(\pi \left(n-1\right)\right)}^{-1}\\ & - & \frac{2r_{+}\left(\frac{-\left(2-2n\right)q^{2}r^{1-2n}}{2\sqrt{1+\frac{q^{2}r^{2-2n}}{\beta^{2}}}\beta^{2}}+\frac{\ln\left(2-2n\right)q^{2}r^{1-2n}}{4\sqrt{1+\frac{q^{2}r^{2-2n}}{\beta^{2}}}\beta^{2}}\right)\beta^{2}}{\pi (n-1)}-(\frac{(n-2)k}{4\pi r_{+}}-\frac{r_{+}\Lambda }{2\pi (n-1)}+(\\ & \times & m^{2}c_{0}){\left(4\pi r_{+}^{3}\right)}^{-1}\;ℸ+\frac{2\beta^{2}r_{+}}{\pi (n-1)})ℶ\left(\frac{d\Delta }{dr_{+}}\right)), \end{array}$$

where $\left(\frac{d\Delta }{dr_{+}}\right)=-\frac{\left(n-1\right)r^{n-2}}{4}-\frac{D}{X^{2}}$,

$$\begin{array}{ccc} D & = & -(2r_{+}^{1-n}\xi (\frac{1}{2}r_{+}^{n-1}(-\frac{k(n-2)}{4\pi r_{+}^{2}}+\frac{m^{2}c_{0}}{4\pi r_{+}^{3}}(3r_{+}^{2}c_{1}+2\left(n-2\right)r_{+}c_{0}c_{2}\\ & + & \left(n-3\right)\left(n-2\right)c_{0}^{2}c_{3})-\frac{3m^{2}c_{0}}{4\pi r_{+}^{4}}ℸ-\frac{\Lambda }{2\pi (n-1)}+\frac{2\beta^{2}r_{+}}{\pi (n-1)}ℶ+(2r_{+}\\ & \times & (-\left(\left(2-2n\right)q^{2}r_{+}^{1-2n}\right){\left(2\sqrt{1+\frac{q^{2}r_{+}^{2-2n}}{\beta^{2}}}\beta^{2}\right)}^{-1}+\left(\left(2-2n\right)q^{2}r_{+}^{1-2n}\right)\\ & \times & {\left(2\left(1+\sqrt{1+\frac{q^{2}r_{+}^{2-2n}}{\beta^{2}}}\right)\sqrt{1+\frac{q^{2}r_{+}^{2-2n}}{\beta^{2}}}\beta^{2}\right)}^{-1})\beta^{2}){\left(\pi \left(n-1\right)\right)}^{-1})\;X\\ & + & \frac{1}{4}\left(n-1\right)r_{+}^{n-2}X^{2})), \end{array}$$

and $X=\left(\frac{k(n-2)}{4\pi r_{+}}+\frac{m^{2}c_{0}}{4\pi r_{+}^{3}}ℸ-\frac{r_{+}\Lambda }{2\pi (n-1)}+\frac{2\beta^{2}r_{+}}{\pi (n-1)}ℶ\right).$

## Appendix B

The enthalpy of BH becomes

$$\begin{array}{ccc} H & = & kr_{+}^{n-2}-\frac{2r_{+}^{n}\Lambda }{n(n-1)}+\frac{8\beta^{2}r_{+}^{n}}{n(n-1)}ℶ+\frac{\left(2n-1\right)\left(1-\sqrt{1+\frac{q^{2}}{\beta^{2}}r_{+}^{2-2n}}\right)}{n})+(8\\ & \times & \left(n-1\right)q^{2}r_{+}^{-2n}){\left(n^{2}\left(n-2\right)\right)}^{-1}F\left(\frac{1}{2},\frac{n-2}{2(n-1)},\frac{4-3n}{2-2n},\frac{-q^{2}r_{+}^{2-2n}}{\beta^{2}}\right)+(\\ & \times & c_{0}m^{2}r_{+}^{n-1})n-1(c_{1}+\frac{\left(n-1\right)c_{0}c_{2}}{r_{+}}+\frac{\left(n-1\right)\left(n-2\right)c_{0}^{2}c_{3}}{r_{+}^{2}}+(\left(n-1\right)(n\\ & - & 2)\left(n-3\right)c_{0}^{3}c_{4}){\left(r_{+}^{3}\right)}^{-1})-\frac{(n-2)k}{4\pi r_{+}}-(\frac{r_{+}\Lambda }{2\pi (n-1)}+\frac{m^{2}c_{0}}{4\pi r_{+}^{3}}\;ℸ+(2\beta^{2}r_{+}\\ & \times & {)\left(\pi \left(n-1\right)\right)}^{-1}ℶ)\Delta (-4\pi r_{+}^{2}(\frac{k(n-2)}{4\pi r_{+}^{2}}+k\left(n-2\right)r_{+}^{n-3}-\frac{\Lambda }{2\pi (n-1)}\\ & - & \frac{2r_{+}^{n-1}\Lambda }{\left(n-1\right)}+(8r_{+}^{n-1}\beta^{2}(\left(\left(2n-1\right)\left(1-{\left(1+\left(q^{2}r_{+}^{2-2n}\right){\left(\beta^{2}\right)}^{-1}\right)}_{\frac{1}{2}}\right)\right){\left(n\right)}^{-1}\\ & + & \frac{1}{2}\ln\left(1+\sqrt{1+\frac{q^{2}r_{+}^{2-2n}}{\beta^{2}}}\right))){\left(n-1\right)}^{-1}+(8r_{+}^{n}\beta^{2}((\ln\left(2n-n\right)q^{2}r_{+}^{1-2n}\\ & \times & ){\left(4\beta^{2}\left(1+\sqrt{1+\frac{q^{2}r_{+}^{2-2n}}{\beta^{2}}}\right)\right)}^{-1}-\frac{\left(2-2n\right)\left(2n-1\right)q^{2}r^{1-2n}}{2n\left(1+\sqrt{1+\frac{q^{2}r_{+}^{2-2n}}{\beta^{2}}}\right)\beta^{2}}))(n(n-1\\ & \times & {))}^{-1}+\left(8\left(2-n\right)\left(n-1\right)q^{2}r_{+}^{1-n}F\left(\frac{1}{2},\frac{n-2}{2(n-1)},\frac{4-3n}{2-2n},\frac{-q^{2}r_{+}^{2-2n}}{\beta^{2}}\right)\right)\\ & \times & {\left(n^{2}\left(n-2\right)\right)}^{-1}-(8\left(2-2n\right)\left(-1+n\right)q^{4}r_{+}^{3-3n}\;F^{1}(\frac{1}{2},\frac{n-2}{2(n-1)},(4-3\\ & \times & {n)\left(2-2n\right)}^{-1},\frac{-q^{2}r_{+}^{2-2n}}{\beta^{2}})){\left(\left(n-2\right)n^{2}\beta^{2}\right)}^{-1}+\frac{c_{0}m^{2}r_{+}^{n-1}}{\left(n-1\right)}(-(\left(n-1\right)\\ & \times & c_{0}c_{2}){\left(r_{+}^{2}\right)}^{-1}-\frac{2\left(n-1\right)\left(n-2\right)c_{0}^{2}c_{3}}{r_{+}^{3}}-\left(3\left(n-1\right)\left(n-2\right)\left(n-3\right)c_{0}^{3}c_{4}\right)(r_{+}^{4}\\ & \times & {)}^{-1})+c_{0}m^{2}r_{+}^{n-2}(c_{1}+\frac{\left(n-1\right)c_{0}c_{2}}{r_{+}}+\frac{\left(n-1\right)\left(n-2\right)c_{0}^{2}c_{3}}{r_{+}^{2}}+(\left(n-1\right)\\ & \times & \left(n-2\right)\left(n-3\right)c_{0}^{3}c_{4}){\left(r_{+}^{3}\right)}^{-1})+\Delta (\frac{k(n-2)}{4\pi r_{+}^{2}}+\frac{\Lambda }{2\pi (n-1)}-(m^{2}c_{0}(3r_{+}^{2}\\ & \times & c_{1}+2\left(n-2\right)r_{+}c_{0}c_{2}+\left(n-3\right)\left(n-2\right)c_{0}^{2}c_{3})){\left(4\pi r_{+}^{3}\right)}^{-1}+\frac{3m^{2}c_{0}}{4\pi r_{+}^{4}}ℸ-(2\\ & \times & \beta^{2}\left(1+\frac{1}{2}\ln(1+\sqrt{1+\frac{q^{2}r_{+}^{2-2n}}{\beta^{2}}}\right)-\sqrt{1+\frac{q^{2}r_{+}^{2-2n}}{\beta^{2}}}{)))\left(\pi \left(n-1\right)\right)}^{-1}\\ & - & (2r_{+}(\left(-\left(2-2n\right)q^{2}r_{+}^{1-2n}\right){\left(2\sqrt{1+\frac{q^{2}r_{+}^{2-2n}}{\beta^{2}}}\beta^{2}\right)}^{-1}+\frac{\ln\left(2-2n\right)q^{2}r_{+}^{1-2n}}{4\sqrt{1+\frac{q^{2}r_{+}^{2-2n}}{\beta^{2}}}\beta^{2}}\\ & \times & )\beta^{2}{)\left(\pi \left(n-1\right)\right)}^{-1}-\left(\frac{(n-2)k}{4\pi r_{+}}-\frac{r_{+}\Lambda }{2\pi (n-1)}+\frac{m^{2}c_{0}}{4\pi r_{+}^{3}}\;ℸ+\frac{2\beta^{2}r_{+}}{\pi (n-1)}\right)\\ & \times & ℶ\left(\frac{d\Delta }{dr_{+}}\right))\left(4\pi r_{+}^{3}\right){\left(3\right)}^{-1}. \end{array}$$

## Appendix C

The relation for logarithmic-corrected Gibbs free energy becomes as follows:

$$\begin{array}{ccc} G & = & kr_{+}^{n-2}-\frac{2r_{+}^{n}\Lambda }{n(n-1)}+\frac{8\beta^{2}r_{+}^{n}}{n(n-1)}ℶ+\frac{\left(2n-1\right)\left(1-\sqrt{1+\frac{q^{2}}{\beta^{2}}r^{2-2n}}\right)}{n})+(8\\ & \times & \left(n-1\right)q^{2}r_{+}^{-2n}){\left(n^{2}\left(n-2\right)\right)}^{-1}F\left(\frac{1}{2},\frac{n-2}{2(n-1)},\frac{4-3n}{2-2n},\frac{-q^{2}r_{+}^{2-2n}}{\beta^{2}}\right)+(c_{0}\\ & \times & m^{2}r_{+}^{n-1}){\left(n-1\right)}^{-1}(c_{1}+\frac{\left(n-1\right)c_{0}c_{2}}{r_{+}}+\frac{\left(n-1\right)\left(n-2\right)c_{0}^{2}c_{3}}{r_{+}^{2}}+(\left(n-1\right)\\ & \times & \left(n-2\right)\left(n-3\right)c_{0}^{3}c_{4}){\left(r_{+}^{3}\right)}^{-1})-\frac{(n-2)k}{4\pi r_{+}}-(\frac{r_{+}\Lambda }{2\pi (n-1)}+\frac{m^{2}c_{0}}{4\pi r_{+}^{3}}\;ℸ+(2\\ & \times & \beta^{2}r_{+}){\left(\pi \left(n-1\right)\right)}^{-1}ℶ)\Delta +(-4\pi r_{+}^{2}(\frac{k(n-2)}{4\pi r_{+}^{2}}+k\left(n-2\right)r_{+}^{n-3}-\left(\Lambda \right)(2\\ & \times & {\pi \left(n-1\right))}^{-1}-\frac{2r_{+}^{n-1}\Lambda }{\left(n-1\right)}+(8r_{+}^{n-1}\beta^{2}(\frac{\left(2n-1\right)(1-{\left(1+\frac{q^{2}r^{2-2n}}{\beta^{2}}\right)}^{\frac{1}{2}})}{n}+\frac{1}{2}\\ & \times & \ln\left(1+{\left(1+\frac{q^{2}r^{2-2n}}{\beta^{2}}\right)}^{\frac{1}{2}}\right))){\left(n-1\right)}^{-1}+(8r_{+}^{n}\beta^{2}(\frac{ln\left(2n-n\right)q^{2}r_{+}^{1-2n}}{4\beta^{2}\left(1+\sqrt{1+\frac{q^{2}r_{+}^{2-2n}}{\beta^{2}}}\right)}\\ & - & \frac{\left(2-2n\right)\left(2n-1\right)q^{2}r^{1-2n}}{2n\left(1+\sqrt{1+\frac{q^{2}r_{+}^{2-2n}}{\beta^{2}}}\right)\beta^{2}})){\left(n\left(n-1\right)\right)}^{-1}+(8\left(2-n\right)\left(n-1\right)q^{2}r_{+}^{1-n}F(\frac{1}{2},\\ & \times & \frac{n-2}{2(n-1)},\frac{4-3n}{2-2n},\frac{-q^{2}r_{+}^{2-2n}}{\beta^{2}})){\left(n^{2}\left(n-2\right)\right)}^{-1}-(8\left(2-2n\right)\left(-1+n\right)q^{4}r_{+}^{3-3n}\\ & \times & \;F^{1}\left(\frac{1}{2},\frac{n-2}{2(n-1)},\frac{4-3n}{2-2n},\frac{-q^{2}r_{+}^{2-2n}}{\beta^{2}}\right)){\left(\left(n-2\right)n^{2}\beta^{2}\right)}^{-1}+\frac{c_{0}m^{2}r_{+}^{n-1}}{\left(n-1\right)}\\ & \times & \left(-\frac{\left(n-1\right)c_{0}c_{2}}{r_{+}^{2}}-\frac{2\left(n-1\right)\left(n-2\right)c_{0}^{2}c_{3}}{r_{+}^{3}}-\frac{3\left(n-1\right)\left(n-2\right)\left(n-3\right)c_{0}^{3}c_{4}}{r_{+}^{4}}\right)\\ & + & c_{0}m^{2}r_{+}^{n-2}(c_{1}+\frac{\left(n-1\right)c_{0}c_{2}}{r_{+}}+\frac{\left(n-1\right)\left(n-2\right)c_{0}^{2}c_{3}}{r_{+}^{2}}+(\left(n-1\right)\left(n-2\right)(n\\ & - & 3)c_{0}^{3}c_{4}){\left(r_{+}^{3}\right)}^{-1})+\Delta (\frac{k(n-2)}{4\pi r_{+}^{2}}+\frac{\Lambda }{2\pi (n-1)}-(m^{2}c_{0}(3r_{+}^{2}c_{1}+2\left(n-2\right)r_{+}\\ & \times & c_{0}c_{2}+\left(n-3\right)\left(n-2\right)c_{0}^{2}c_{3})){\left(4\pi r_{+}^{3}\right)}^{-1}+\frac{3m^{2}c_{0}}{4\pi r_{+}^{4}}ℸ-(2\beta^{2}(1+\frac{1}{2}\ln(1+(1\\ & + & \frac{q^{2}r_{+}^{2-2n}}{\beta^{2}}{)}^{\frac{1}{2}})-{\left(1+\left(q^{2}r_{+}^{2-2n}\right){\left(\beta^{2}\right)}^{-1}\right)}^{{}^{\frac{1}{2}}}))){\left(\pi \left(n-1\right)\right)}^{-1}-(2r_{+}((-(2-2\\ & \times & n)q^{2}r_{+}^{1-2n}){\left(2\sqrt{1+\frac{q^{2}r_{+}^{2-2n}}{\beta^{2}}}\beta^{2}\right)}^{-1}+\frac{\ln\left(2-2n\right)q^{2}r_{+}^{1-2n}}{4\sqrt{1+\frac{q^{2}r_{+}^{2-2n}}{\beta^{2}}}\beta^{2}})\beta^{2}){\left(\pi \left(n-1\right)\right)}^{-1}\\ & - & \left(\frac{(n-2)k}{4\pi r_{+}}-\frac{r_{+}\Lambda }{2\pi (n-1)}+\frac{m^{2}c_{0}}{4\pi r_{+}^{3}}\;ℸ+\frac{2\beta^{2}r_{+}}{\pi (n-1)}\right)ℶ\left(\frac{d\Delta }{dr_{+}}\right)\left(\frac{4\pi r_{+}^{3}}{3}\right)))\\ & \times & \left(4\pi r_{+}^{3}\right){\left(3\right)}^{-1} \end{array}$$

## Appendix D

The specific heat of BH takes the following form

$$\begin{array}{ccc} C & = & \frac{(n-2)k}{4\pi r_{+}}-\frac{r_{+}\Lambda }{2\pi (n-1)}+\frac{m^{2}c_{0}}{4\pi r_{+}^{3}}\;ℸ+\frac{2\beta^{2}r_{+}}{\pi (n-1)}ℶ((-\frac{\left(n-1\right)r_{+}^{n-2}}{4}-\log\xi \\ & \times & (\frac{k{\left(n-2\right)}^{2}r_{+}^{n-3}}{16\pi }+\frac{\left(1+\frac{1}{2}\ln\left(1+\sqrt{1+\frac{q^{2}r_{+}^{2-2n}}{\beta^{2}}}\right)-\sqrt{1+\frac{q^{2}r_{+}^{2-2n}}{\beta^{2}}}\right))\beta^{2}}{2\pi (n-1)}\\ r & - & \frac{\Lambda }{8\pi (n-1)}+\frac{m^{2}c_{0}r_{+}^{n-5}\left(n-4\right)}{16\pi }ℸ+(m^{2}c_{0}r_{+}^{n-4}(3r_{+}^{2}c_{1}+2\left(n-2\right)r_{+}c_{0}c_{2}\\ & + & \left(n-3\right)\left(n-2\right)c_{0}^{2}c_{3}{))\left(16\pi \right)}^{-1}+(r_{+}\beta^{2}\left(1+\frac{1}{2}\ln(1+\sqrt{1+\frac{q^{2}r_{+}^{2-2n}}{\beta^{2}}}\right)\\ & - & \sqrt{1+\frac{q^{2}r_{+}^{2-2n}}{\beta^{2}}}))){\left(2\pi \left(n-1\right)\right)}^{-1}(\frac{k\left(n-2\right)r_{+}^{n-2}}{16\pi }-\frac{r_{+}\Lambda }{8\pi (n-1)}+(m^{2}c_{0}\\ & \times & r_{+}^{n-4}{)\left(16\pi \right)}^{-1}ℸ+\frac{r_{+}\beta^{2}\left(1+\frac{1}{2}\ln(1+\sqrt{1+\frac{q^{2}r_{+}^{2-2n}}{\beta^{2}}}\right)-\sqrt{1+\frac{q^{2}r_{+}^{2-2n}}{\beta^{2}}}))}{2\pi (n-1)}\\ & \times & )))((\frac{k(n-2)}{4\pi r_{+}^{2}}+\frac{\Lambda }{2\pi (n-1)}-(m^{2}c_{0}(3r_{+}^{2}c_{1}+2\left(n-2\right)rc_{0}c_{2}+(n-3\\ & \times & )\left(n-2\right)c_{0}^{2}c_{3})){\left(4\pi r_{+}^{3}\right)}^{-1}+\frac{3m^{2}c_{0}}{4\pi r_{+}^{4}}ℸ-(2\beta^{2}(1+\frac{1}{2}\ln(1+{\left(1+\frac{q^{2}r_{+}^{2-2n}}{\beta^{2}}\right)}^{\frac{1}{2}}\\ & \times & )-\sqrt{1+\frac{q^{2}r_{+}^{2-2n}}{\beta^{2}}}{)))\left(\pi \left(n-1\right)\right)}^{-1}-(2r_{+}(\frac{-\left(2-2n\right)q^{2}r_{+}^{1-2n}}{2\sqrt{1+\frac{q^{2}r_{+}^{2-2n}}{\beta^{2}}}\beta^{2}}+(\ln(2\\ & - & 2n)q^{2}r_{+}^{1-2n}){\left(4\sqrt{1+\frac{q^{2}r_{+}^{2-2n}}{\beta^{2}}}\beta^{2}\right)}^{-1})\beta^{2}){\left(\pi \left(n-1\right)\right)}^{-1}{)}^{-1}. \end{array}$$
